# Providing quality end-of-life care to older people in the era of COVID-19: perspectives from five countries

**DOI:** 10.1017/S1041610220000836

**Published:** 2020-05-11

**Authors:** Maria I. Lapid, Raymond Koopmans, Elizabeth L. Sampson, Lieve Van den Block, Carmelle Peisah

**Affiliations:** 1 Department of Psychiatry and Psychology, Mayo Clinic, Rochester, MN, USA; 2 Department of Primary and Community Care, Radboud University Medical Center, Nijmegen, The Netherlands; 3 Joachim en Anna, Center for Specialized Geriatric Care, Nijmegen, The Netherlands; 4 Marie Curie Palliative Care Research Department, Division of Psychiatry, UCL, London, UK; 5 Barnet, Enfield and Haringey Mental Health Liaison Service, North Middlesex University Hospital NHS Trust, London, UK; 6 Aging and Palliative Care, VUB-UGhent End-of-Life Care Research Group, Vrije Universiteit Brussel (VUB), Department of Family Medicine and Chronic Care, Brussels, Belgium; 7 University New South Wales, Kensington, Australia; 8 Sydney University, Sydney, Australia; 9 Capacity Australia, Sydney, Australia

## Introduction

Providing quality end-of-life care for older people is one of our biggest challenges in this new COVID-19 era. Advanced age, because of its association with a range of physical comorbidities, is associated with greater mortality with COVID-19. Specifically, case fatality rates in the 70+ age group range from 8.6% to 13.4% compared with 0.0026–0.3% in those under 45 (Ruan, 2020; Zhou *et al.*, 2020). However, vulnerability is not conferred by physiological factors alone, but additionally by psychosocial factors such as ageism and ethical considerations such as distributive justice (Stirling, 2020; Truog *et al.*, 2020). Those who are at high risk for COVID-19 are the same patients we take care of in geriatric psychiatry, geriatrics, nursing home, hospice, and palliative care, that is, older, ill, frail, cognitively impaired, at high risk of delirium with mental and physical comorbidities (van den Brink *et al.*, 2017). An acute COVID infection may be, as Ballentine (2020) suggested, “what collapses the house of cards” for our vulnerable patients.

In ensuring optimal end-of-life care for this vulnerable population, the palliative and hospice care philosophy is critical. This philosophy is based on the alleviation of distressing pain and other burdensome symptoms, provision of support for psychosocial and spiritual issues and the optimization of quality of life for patients with life-limiting illnesses and their families or caregivers (Ballentine, 2020). Ensuring comfort in dying, managing symptoms, ensuring high-quality medico-ethical decision-making based on wishes and preferences and supporting families and providers is business as usual for palliative care, which has never been more important.

This paper will explore the key challenges currently afoot in providing quality end-of-life care to older people in the COVID pandemic from the perspective of clinicians and researchers from five countries. We explore ways to preserve the same level of care our patients already receive and to avoid care disparities at the end of life, concluding with suggestions for clinicians trying to achieve this.

## Human rights and bioethical principles at stake

In the pre-COVID-19 era, equitable access to high-quality health, implicitly including end of life (Peisah, 2018), was a human right articulated under Article 25 of the Convention of the Rights of Persons with Disabilities (CRPD) (United Nations Committee on the Rights of Persons With Disabilities, 2020). Similarly, equitable access to palliative care, including pain relief, is a human right owed to all, including those in residential care (Froggatt *et al.*, 2020; Markham *et al.*, 2019; Palliative Care for Older People in Care and Nursing Homes in Europe Project, 2019). The challenge in this COVID-19 era is to provide—where possible given our severely compromised health environments—this equitable access to high-quality death and dying even to the most vulnerable.

To honor ethical principles of beneficence, non-maleficence, preserving autonomy, and truth-telling, clinicians must act in the best interests of their patients in a transparent manner to maximize autonomous decision-making and to do no harm. For many of us depending on health care settings and resources, this pandemic has forced our hand and limited certain treatment options. Accordingly, we cannot offer choice where choice does not exist (e.g. “Do you want to go to Intensive Care [ICU]?”) as advocated in Australia (Greenaway and Arunarthy, 2020). Ballentine stated that maintaining grace, while helping patients and families to understand and decide, is all in palliative care’s “wheelhouse” (Ballentine, 2020).

There have been a number of ethical approaches to resource allocation, including “first come, first served,” saving most life years or maximizing benefits and prognosis, giving priority to the worst off, or to those able to contribute most such as health workers (Emanuel *et al.*, 2020). Additionally, we have a duty of resource stewardship enabling us to “do the best for the most.” Age should not be a criterion per se among any of these options but tends to correlate with poorer prognosis, less life years, and less—or no—benefits of invasive treatments. We know that both frailty and age are associated with worst non-beneficial outcomes in intensive care (Carter *et al.*, 2019; Muscedere *et al.*, 2017), and frailty scoring has become a cornerstone of COVID-19 ICU resource allocation in many countries, although this remains controversial in some parts of the world (see below).

The primary role of beneficence and non-maleficence in resource allocation existed pre-COVID. At a National Academies workshop in November 2019 to discuss Crisis Standards of Care principles and processes for non-disaster shortages (Hick *et al.*, 2009; Hick *et al.*, 2020), strategies for scarce resource situations included “Reallocate” (removal of resources such as ventilators and Extra-Corporeal Membrane Oxygenation from one area or patient and allocation to those with higher likelihood of benefit). Therefore, in the allocation process, the older vulnerable person who is unlikely to benefit from ventilation is most likely to be diverted—but beneficently so—to palliative care. Understanding that such decision-making is driven by principles of beneficence may protect clinicians from the moral injury and distress (Greenberg *et al.*, 2020; Williamson *et al.*, 2020) incurred by resource-based ventilator rationing (Truog *et al.*, 2020). Moreover, if older people are diverted to palliative care for beneficent reasons, this is the message we wish to convey to patients and families.

Given this reality in the middle of a pandemic, we as providers need to be prepared, and we need to prepare patients and families. We need to ensure we are honoring patients’ wishes, while also ensuring that they receive the appropriate level of care and treatment they need and desire. There is room despite this pandemic to honor the human right to expression of will and preferences, as articulated in Article 12, CRPD. In having conversations about goals of care, and preferences regarding code status, resuscitation, intubation, avoiding the Emergency Department or ICU, or other aggressive interventions associated with low likelihood of meaningful recovery, it is critical to let patients and proxies know what to expect.

To address these morally loaded and very delicate issues, the Dutch Association of Elderly Care Physicians, General Practitioners, Geriatricians, and Internal Medicine specialists issued on March 27, 2020, a practice guideline for triage decisions regarding admission of frail elderly with COVID-19 infection (or suspected) to hospitals, ICUs, or to offer home treatment. Advantages (e.g. more advanced treatment options including ICU care and medications) and disadvantages (e.g. increased risk of delirium, isolation, and functional decline) of hospitalization are outlined. It is recommended that treating physicians consider the prospects of rehabilitation following often burdensome hospitalization. It is well known that sarcopenia associated with prolonged hospital and ICU admission and mechanical ventilation results in mobility loss and functional decline often requiring intensive geriatric rehabilitation lasting up to 12 months. Negative respiratory effects of mechanical ventilation, provisionally called Corona Obstructive Lung Disease, and potential exacerbation of pre-existing cognitive problems further add to the futility of ICU treatment. In order to assist decision-making regarding hospitalization, the guideline suggests using the Clinical Frailty Scale (CFS, Table 1) (Rockwood *et al.*, 2005). Those with scores between 6 (moderately frail) and 9 (terminally ill; mostly those who live in nursing homes) will not have enough reserve capacity to survive and will probably not benefit from hospital care. The benefits are unclear in those with CFS 4 or 5. However, shared decision-making is always warranted with respect to personal wishes and preferences of the patient and proxies.

**Table 1:** Clinical Frailty Scale (Rockwood *et al.*, 2005), reprinted with permission

The Canadian Study of Health and Aging Clinical Frailty Scale
1 *Very fit*—robust, active, energetic, well motivated, and fit; these people commonly exercise regularly and are in the most fit group for their age
2 *Well*—without active disease, but less fit than people in category 1
3 *Well, with treated comorbid disease*—disease symptoms are well controlled compared with those in category 4
4 *Apparently vulnerable*—although not frankly dependent, these people commonly complain of being “slowed up” or have disease symptoms
5 *Mildly frail*—with limited dependence on others for instrumental activities of daily living
6 *Moderately frail*—help is needed with both instrumental and non-instrumental activities of daily living
7 *Severely frail*—completely dependent on others for the activities of daily living or terminally ill

Note: In 2007, the CFS© was expanded from a seven-point scale to the present nine-point scale.

A similar process has been developed in the United Kingdom (UK) where the National Institute for Clinical Excellence has released guidance on admission to hospitals and ICU highlighting the importance of the CFS, taking a holistic approach to considering comorbidities and underlying health conditions. An initial suggestion that a CFS score of 5 be used as part of the decision-making process for ICU admission was met with justified objections from a range of stakeholders including organizations representing people with learning difficulties, dementia, and mental health problems. This led to the guidance being caveated with the statement “The CFS should not be used in younger people, people with stable long-term disabilities (e.g. cerebral palsy), learning disabilities, or autism. An individualized assessment is recommended in all cases where the CFS is not appropriate.” Further national government guidance has not been issued, the suggestion being that these decisions are made and monitored at a local level. All acute hospitals in the UK have now established ethics panels to review individual cases and support staff making these difficult decisions.

In Belgium, a COVID-19 guideline for symptom control and clinical decision-making for nursing homes has been released (latest update March 25, 2020), endorsed by the Belgian Association for Gerontology and Geriatric and Crataegus (the Flemish organization responsible for education of the Coordinating and Advisory Physicians working in nursing homes). The document includes guidelines for symptom management specific to COVID-19 and risk-benefit analyses regarding hospitalization of nursing home residents, taking into account current ethical recommendations. Consistent with Australia, the Netherlands, and the UK, there are strong recommendations to use the CFS to guide decision-making, albeit using different cut-off scores. Specifically, residents with CFS scores of 8 or 9 should receive palliative care in the nursing home unless advance care planning (ACP) indicates otherwise. For those with a score of 7, consultation should occur with the resident and/or family regarding palliative or supportive care in the nursing home, or supportive care in an acute geriatric hospital unit. Those with CFS scores less than 7 and two or more alarming symptoms (e.g. consciousness, respiratory rate, saturation level, and pulse/blood pressure) are recommended for hospital admission, taking into account ACP, wishes, and preferences, in consultation with the Emergency Department.

Included in this Belgian guidance is ethical advice from the Belgian Society of Intensive Care Medicine concerning proportionality of critical care during the pandemic. This clearly states that “intensive care medicine should be reserved for patients in whom a good or at least acceptable outcome can be expected, after hospital discharge.” It highlights the need for triage if there is insufficient ICU capacity, although in Belgium so far this has not been the case, as ICU capacity has more than doubled to ensure sufficient capacity. Regarding age, the advice reads “although an increased age is associated with worse outcomes in COViD-19, age in isolation cannot be used for triage decisions, but should be integrated with other clinical parameters.” It recommends early initiation of ACP (before ICU admission and before an acute phase) and advises against ICU admission for nursing home residents unless there is a clearly defined realistic therapeutic goal.

## Advance care planning

ACP is vital in the COVID-19 context because of the unpredictable and rapid deterioration seen in some patients, leaving little time to consult with the patient or family. ACP is a particularly pressing issue for people with dementia who may lack capacity to make these decisions for themselves.

Although harder to do in a pandemic, escalation and facilitation of ACP implementation has been the cornerstone of the pandemic response for those countries such as Australia afforded the luxury of some preparation (Advance Care Planning Australia, 2020). In Dutch long-term care facilities, elderly care physicians with a 3-year specialist training program have long-lasting experiences with ACP conversations (Koopmans *et al.*, 2017). For every newly admitted resident, preferences and wishes regarding resuscitation, hospital admission, ICU treatment, and the existence of an ACP are established. Preferences are recorded in the medical file and regularly evaluated and adapted. In the COVID era, these discussions must be adapted since prognosis of hospitalization and ICU treatment are futile in frail elderly. In the Dutch experience, most older people agree not to be hospitalized and prefer to stay in their home environment. In Belgium, there has been an increase in ACP over the past few years in nursing homes, but there is still room for much improvement (Gilissen *et al.*, 2018).

To facilitate ACP, just as some advocate the use of the Surprise Question in dementia (“Would you be surprised if this patient were to die in the next 6–12 months”) (Empowered Project, 2020; Markham *et al.*, 2019; White *et al.*, 2017), adjunctive use of the Supportive and Palliative Care Indicators Tool (Highet *et al.*, 2014) has been advocated in the COVID-19 pandemic to promote transparency and truthfulness in advance planning (Greenaway and Arunarthy, 2020).

One important consideration concerning ACP is that for people who have not had the opportunity to discuss and reflect on their wishes and preferences for end-of-life decisions pre-COVID, ACP might be biased toward the emotions and fears induced by the pandemic. Making advance directives without in-depth reflection and opportunities to discuss might be very difficult for people. Hence, sensitive and compassionate communication seem key, and organizations such as Center to Advance Palliative Care in the USA have been making and distributing communication guidelines that might help with this. Capacity issues associated with impaired cognition and delirium complicate this further.

This pandemic has unfolded a lack of awareness among people about end-of-life decision-making. At least in Belgium, where euthanasia and palliative sedation are the two end-of-life practices that have received most attention in public forums over the past two decades, the lack of clarity around other end-of-life decisions such as non-treatment or withholding treatment (e.g. do not hospitalize or do not intubate) is surprising. The crisis is an opportunity to discuss these issues more openly and increase death literacy in the population, hopefully culminating in more authentic ACP in the future.

## Symptom management including palliative sedation

In the Netherlands, the Dutch Association of Elderly Care Physicians issued treatment guidelines regarding the most prevalent symptoms in COVID patients such as dyspnea, pain, cough, delirium, nausea and vomiting, anxiety, and sleepiness. Similarly, in Belgium, the aforementioned nursing home document provides guidelines for pharmacological and non-pharmacological approaches to symptom management in line with existing guidelines for symptom management in palliative care. Treatment of dyspnea, especially the so-called silent dyspnea, with a saturation level of 70% without visible signs, is an additional challenge. In these cases, treatment with oxygen supplemented by morphine is warranted. However, Dutch and Belgian long-term care facilities face shortages in oxygen supplies, which is a serious threat for optimal symptom management.

Additionally, there is often a need to apply palliative sedation. Palliative sedation involves the deliberate lowering of the patient’s consciousness in the final stage of life in order to relieve “refractory symptoms” that cause unbearable suffering if conventional modes of treatment are ineffective or do not act quickly enough. Although there are no research data yet, it is expected that refractory dyspnea with concomitant anxiety and delirium in patients with dementia may require palliative sedation. There is also a risk of shortages of medication, especially midazolam, which is the first-choice benzodiazepine for palliative sedation. Guidelines advise diazepam or lorazepam as alternatives in such cases.

Real and potential shortages of medication, oxygen supplies, and opioid driver pumps are relevant to all countries. As such, equal to the importance of ensuring adequate personal protective equipment (PPE) and ventilators, is the need to ensure an ongoing supply of equipment required to provide quality palliative care.

## Voluntary-assisted dying and euthanasia

If ever there was time for careful scrutiny of decision-making with regard to voluntary-assisted dying (VAD), now is the time. Older people’s perceptions of being a burden will be ever more salient, compounding depression, hopelessness, and loneliness, all of which may fuel VAD requests (Peisah *et al.*, 2019; Wand *et al.*, 2018).

So far, in the Netherlands, Belgium, and Australia, there are no signs of changing either VAD or euthanasia practice. Legislation remains the same. However, since the Corona crisis, the Netherlands’ center of expertise on euthanasia has closed. It is not clear what the impact of this will be, but there may be a risk to those desperately seeking euthanasia who may resort to other alternatives. There are some anecdotes that some people purposively want to be infected by COVID in order to hasten death.

In Belgium, there are currently no specific debates on euthanasia. Discussions center around non-treatment decisions, particularly whether age will be used as a triage for hospital or ICU admission. While current guidelines are clear that this is not the case, there is a lot of confusion in the media and public opinion, misinterpreting the intent that proportionate and dignified care is the aim for all. This is compounded by increasing outbreaks in long-term care facilities precipitating panic among the sector regarding insufficient resources and personnel to deal with the pandemic. These are urgent issues to address in Belgium.

## Long-term care facilities (nursing or care homes)

Much of the care of older persons, particularly those with dementia, lies with long-term care facilities. Older patients approaching the end of life can be managed entirely in long-term care facilities without compromising either quality of care or survival (Hui *et al.*, 2014). The Dutch long-term care sector is fast transitioning former residential homes or other wards to either specific CORONA wards, with quarantine or hospice wards, to provide care for people admitted from their homes or discharged from hospitals. This is a major organizational operation.

On March 12, 2020, the Flemish government in Belgium, and on March 19, 2020, the Dutch government in the Netherlands excluded entry of family members from nursing homes as has been the case in Australia, the USA, and the UK. In the UK, the notion of more stringent “shielding” has been implemented for those considered extremely vulnerable, that is people undergoing chemotherapy for cancer or severe chronic obstructive pulmonary disease.

Exceptions to family visits are sometimes made for residents in a terminal stage; however, even then numbers of family members are highly restricted and touching infected residents precluded. There are now many stories of people dying alone or saying goodbye via iPads, the impact on residents, and on bereaved families clearly evident. When patients die, the inability to go to a place of worship or gather for funerals makes it harder for families and communities to mourn and grieve.

These restrictions are placing an enormous toll on people with dementia who are isolated, socially disconnected, and do not understand COVID-imposed restrictions, culminating in challenging behaviors. Apart from unmet need from loneliness and need for intimacy, the sight of staff in PPE can be misinterpreted and frightening. Additionally, imposing social distancing including restriction to rooms is almost impossible to enforce among those with dementia. Notably, in the UK, the government released new guidance on Deprivation of Liberty safeguards that may be implemented to protect persons with dementia whose behavior (i.e. wandering) is putting themselves at risk of contracting COVID-19.

Around the world, solutions to these isolating measures have been to find alternative modes of communication. There has been an enormous increase in using technology for video conferencing in long-term care. Open air concerts, at the front doors and windows of facilities, have been provided by spontaneous actions of volunteers. There are now also numerous technologies and multimedia options to allow remote attendance of funerals and memorials.

Professional caregivers equally require support. Working in the COVID crisis is extremely burdensome and causes stress and anxiety, as does the moral distress associated with watching residents die who may not have otherwise died. Where available, facility psychologists offer support if needed and human resource departments have provided support tools such as online yoga courses. An additional focus of concern has been adequate provision of PPE for and testing of staff of long-term facilities, often neglected in the rush to mobilize and support hospital-based services. Notably, on April 6, 2020, the Dutch Government initiated extra testing of health care professionals who work outside hospital settings. However, in Belgium, thus far the nursing home sector has received less attention than in the Netherlands, at least compared with the hospital and ICU sector. This is further aggravated by the fact that competencies are split across the national and regional governments in Belgium, making it difficult to make joint policies.

In Belgium, as with Australia (Royal Commission into Aged Care Quality and Safety, 2020), COVID-19 has exploded on a background of long-standing neglect of the nursing home sector and need for greater investment in such. In times of pandemics such as this, the weakest part of the health care system will be affected most. In Belgium, as in many countries in the world, care assistants are more present than nurses, and palliative care competences of staff could be improved (Froggatt *et al.*, 2017; Smets *et al.*, 2018). Notably in Belgium, a new Taskforce has been set up by the Flemish government (April 9, 2020), the innovative goals of which are to, among others, implement strategies to exchange personnel between different settings including hospitals and nursing homes.

## Hospice and in-home dying

Hospice programs continue in the pandemic, but the provision of hospice care has drastically changed. In some countries such as the UK where hospice care is predominantly provided by the charity sector, there are great concerns about how the loss of fundraising revenue may lead to closure of clinical services at a time of greatest need.

In the USA, the bulk of hospice care is provided in the home setting. Community palliative care in the home is also available in Australia. We have previously advocated for the choice for those with dementia to remain at home, and die at home, or not (Sorinmade *et al.*, 2018). Notably, shifting hospice and palliative care resources to the community was a key finding in a recent review to inform practice in the pandemic (Etkind *et al.*, 2020). In the USA, multidisciplinary hospice team members (physicians, nurses, nurses’ aides, pharmacists, social workers, chaplains, integrative therapists and volunteers) visit the patient and family in the patient’s place of residence. Recognizing increased risk for both patient/family and hospice staff, internal protocols for hospice agencies now follow guidelines issued by the US Centers for Health and Human Services (Centers for Medicare and Medicaid Services) to protect health care workers who operate in the home and community from COVID-19, including identifying at-risk individuals, screening procedures, and PPE. In the USA, statutory requirement for face-to-face encounters between patient and physicians to qualify for Medicare home health care has been waived and can now be conducted using telehealth (Centers for Medicare & Medicaid Services, 2020).

Dutch high-care hospices, most of which are part of larger nursing home care organizations, keep on running. These hospices or palliative care units within long-term care facilities are predominantly run by elder care physicians with 2-year additional training in palliative care. In Belgium, specialist palliative home care teams, largely funded by the government, are now providing most care via teleconsultations and are visiting patients less often at home or in the nursing homes.

The impact of necessary social exclusion applies equally, if not more, to hospice care. While telehealth can allow patients and providers to remain connected, access to technology may not be feasible for patients and caregivers, and this may add to stress and anxiety, particularly for those who need guidance with medications and other care strategies to manage end-of-life symptoms at home.

With regard to funding of such services, in the US Medicare pays for evaluation, management, and service provision by a physician or nurse practitioner, including payment for a number of non-face-to-face services such as care management services, remote patient monitoring services, and communication technology-based services. In Australia, new Medicare Benefits Schedule items have been created to facilitate telehealth provision. Made available to general practitioners, psychiatrists and other medical practitioners, nurse practitioners, and allied health providers, they are particularly suited to provision of home-based, nursing home, and hospice care. Dutch health insurance companies and the Dutch Government have guaranteed that all additional services and staffing will be reimbursed, stating that none of the long-term care facilities nor hospitals have to face bankruptcy.

## Suggestions: How clinicians can provide quality care to our older patients in the COVID-19 pandemic

1. Follow guidelines to flatten the curve of the disease including handwashing, social distancing, appropriate use of PPE, and maintaining screening and testing protocols (World Health Organization, 2020).
2. Embrace innovation such as telemedicine as an alternative to face-to-face visits to minimize disruption of care and services provided to patients, families, and caregivers.
3. Back to the drawing board: use biopsychosocial approaches to manage psychiatric conditions such as anxiety, depression, cognitive decline, and alcohol misuse.
4. Change work practices to manage workforce demand and supply such as dividing work between physicians that provide care to patients with COVID, and alternate, providing respite and support to each other. Have a low threshold for testing clinicians if the organization can support this.
5. Permanently adapt and refine procedures and treatment protocols based on new evidence and recommendations of professional organization; ensure organizational planning for maintenance of PPE and palliative care resources such as oxygen supplies, medication, and opioid driver pumps.
6. Play an important role in staff support, organizational, and crisis management teams of facilities.
7. Strengthen cooperation and transmural collaboration between primary/community, hospital, and long-term care settings.
8. Be acutely conscious of the acute severing of social connectedness caused by COVID-19 at the most crucial time of end of life. Facilitate continuing social support of patients at the end of life using remote technologies.
9. Encourage ACP based on informed and shared decision making using remote technologies and other online resources (Empowered Project, 2020).
10. Prepare psychosocial and spiritual support for after the current crisis, for health care staff and family members of people who died during this COVID-19 period.

## References

[ref1] Advance Care Planning Australia (2020). COVID-19: Is it time for Australians to make future medical decisions? Available at: https://www.advancecareplanning.org.au/get-involved/read-the-latest-advance-care-planning-news/article/2020/03/23/covid-19-is-it-time-for-australians-to-make-future-medical-decisions#/; accessed 3 April 2020.

[ref2] Ballentine, J. M. (2020). The Role of Palliative Care in a COVID-19 Pandemic (blog). The California State University Shiley Institute for Palliative Care. Available at: https://csupalliativecare.org/palliative-care-and-covid-19/; accessed 3 April 2020.

[ref3] Carter, H. E. et al. (2019). Factors associated with non-beneficial treatments in end of life hospital admissions: a multicentre retrospective cohort study in Australia. BMJ Open, 9, e030955. doi: 10.1136/bmjopen-2019-030955 PMC6858125 31690607

[ref4] Centers for Medicare & Medicaid Services (2020). Coronavirus Disease 2019. Baltimore, MD: U.S. Centers for Medicare & Medicaid Services Available at https://www.cms.gov/; accessed 3 April 2020

[ref5] Emanuel, E. J. et al. (2020). Fair allocation of scarce medical resources in the time of Covid-19. New England Journal of Medicine, doi: 10.1056/NEJMsb2005114 32202722

[ref6] Empowered Project (2020). End of Life Video. Capacity Australia.

[ref7] Etkind, S. N. et al. (2020 (in press)). The role and response of palliative care and hospice services in epidemics and pandemics: a rapid review to inform practice during the COVID-19 pandemic. Journal of Pain and Symptom Management, doi: 10.1016/j.jpainsymman.2020.03.029 PMC7141635 32278097

[ref8] Froggatt, K. et al. (2017). Palliative care development in European care homes and nursing homes: application of a typology of implementation. Journal of the American Medical Directors Association, 18, 550e557–550e514. doi: 10.1016/j.jamda.2017.02.016 PMC5754324 28412166

[ref9] Froggatt, K. A. , Moore, D. C. , Van den Block, L. , Ling, J. , Payne, S. A. and Pace consortium collaborative authors on behalf of the European Association for Palliative Care (2020). Palliative care implementation in long-term care facilities: European Association for Palliative Care White Paper. Journal of the American Medical Directors Association, doi: 10.1016/j.jamda.2020.01.009 32115370

[ref10] Gilissen, J. et al. (2018). How to achieve the desired outcomes of advance care planning in nursing homes: a theory of change. BMC Geriatrics, 18, 47. doi: 10.1186/s12877-018-0723-5 29444645 PMC5813418

[ref11] Greenaway, S. and Arunarthy, S. (2020). Clinical Ethics and Sound Clinical Decision Making in the Context of the Covid19 Pandemic. Western Sydney Local Health District (personal communication).

[ref12] Greenberg, N. , Docherty, M. , Gnanapragasam, S. and Wessely, S. (2020). Managing mental health challenges faced by healthcare workers during covid-19 pandemic. BMJ, 368, m1211. doi: 10.1136/bmj.m1211 32217624

[ref13] Hick, J. L. , Barbera, J. A. and Kelen, G. D. (2009). Refining surge capacity: conventional, contingency, and crisis capacity. Disaster Medicine and Public Health Preparedness, 3, S59–S67. doi: 10.1097/DMP.0b013e31819f1ae2 19349869

[ref14] Hick, J. L. , Hanfling, D. , Wynia, M. K. and Pavia, A. T. (2020). Duty to Plan: Health Care, Crisis Standards of Care, and Novel Coronavirus SARS-CoV-2. NAM Perspectives (discussion paper). Washington, DC: National Academy of Medicine. 10.31478/202003b PMC8406582 34532682

[ref15] Highet, G. , Crawford, D. , Murray, S. A. and Boyd, K. (2014). Development and evaluation of the Supportive and Palliative Care Indicators Tool (SPICT): a mixed-methods study. BMJ Support Palliat Care, 4, 285–290. doi: 10.1136/bmjspcare-2013-000488 24644193

[ref16] Hui, E. et al. (2014). A new model for end-of-life care in nursing homes. Journal of the American Medical Directors Association, 15, 287–289. doi: 10.1016/j.jamda.2013.11.019 24508325

[ref17] Koopmans, R. , Pellegrom, M. and van der Geer, E. R. (2017). The Dutch move beyond the concept of nursing home physician specialists. Journal of the American Medical Directors Association, 18, 746–749. doi: 10.1016/j.jamda.2017.05.013 28668662

[ref18] Markham, S. , Jessop, T. and Peisah, C. (2019). Death, dying and dementia – a pivotal role for the GP. Med Today, 20, 10–17.

[ref19] Muscedere, J. et al. (2017). The impact of frailty on intensive care unit outcomes: a systematic review and meta-analysis. Intensive Care Med, 43, 1105–1122. doi: 10.1007/s00134-017-4867-0 28676896 PMC5501903

[ref20] Palliative Care for Older People in Care and Nursing Homes in Europe (PACE) Project (2019). PALLIATIVE CARE FOR DIGNITY IN OLD AGE: Addressing the Needs of Older People in Long-Term Care Facilities in Europe. Available at: http://www.eupace.eu/publication/pace-policy-recommendations-palliative-care-dignity-old-age; accessed 10 April 2020.

[ref21] Peisah, C. (2018). The palliative psychiatrist: end of life care in the nursing home (abstract). World Psychiatric Association. Thematic Congress, Innovation in Psychiatry: Effective Interventions for Health and Society Melbourne, VIC, Australia.

[ref22] Peisah, C. , Sheahan, L. and White, B. P. (2019). Biggest decision of them all – death and assisted dying: capacity assessments and undue influence screening. Internal Medicine Journal, 49, 792–796. doi: 10.1111/imj.14238 30693625

[ref23] Rockwood, K. et al. (2005). A global clinical measure of fitness and frailty in elderly people. CMAJ, 173, 489–495. doi: 10.1503/cmaj.050051 16129869 PMC1188185

[ref24] Royal Commission into Aged Care Quality and Safety (2020). Interim Report. Commonwealth of Australia. Available at: https://agedcare.royalcommission.gov.au/publications/Pages/interim-report.aspx; accessed 10 April 2020.

[ref25] Ruan, S. (2020). Likelihood of survival of coronavirus disease 2019 (comment, published online). Lancet Infectious Diseases, doi: 10.1016/S1473-3099(20)30257-7 PMC7156221 32240633

[ref26] Smets, T. et al. (2018). The palliative care knowledge of nursing home staff: The EU FP7 PACE cross-sectional survey in 322 nursing homes in six European countries. Palliative Medicine, 32, 1487–1497. doi: 10.1177/0269216318785295 29972343 PMC6158686

[ref27] Sorinmade, O. A. , Peisah, C. and Jackman, J. (2018). Remaining at home with dementia: not ‘one size fits all’. *Psychiatric Bulletin*, eLetter, 26 April 2018. https://www.cambridge.org/core/journals/psychiatric-bulletin/article/new-procedure-for-submitting-letters/C32BBDBC94C609037261C8A97D3BB73C#fndtn-comments

[ref28] Stirling, C. (2020). Re: Covid-19: control measures must be equitable and inclusive (letter to Editor). BMJ, 368. 10.1136/bmj.m1141 32198146

[ref29] Truog, R. D. , Mitchell, C. and Daley, G. Q. (2020). The toughest triage – allocating ventilators in a pandemic. New England Journal of Medicine. doi: 10.1056/NEJMp2005689 32202721

[ref30] United Nations Committee on the Rights of Persons With Disabilities (2020). Convention on the Rights of Persons with Disabilities. United Nations: Office of the High Commissioner, Human Rights. Available at: https://www.ohchr.org/EN/HRBodies/CRPD/Pages/ConventionRightsPersonsWithDisabilities.aspx; accessed 9 April 2020.

[ref31] van den Brink, A. M. A. , Gerritsen, D. L. , de Valk, M. M. H. , Oude Voshaar, R. C. and Koopmans, R. (2017). Characteristics and health conditions of a group of nursing home patients with mental-physical multimorbidity – the MAPPING study. International Psychogeriatric, 29, 1037–1047. doi: 10.1017/S1041610217000230 28260543

[ref32] Wand, A. P. F. , Peisah, C. , Draper, B. and Brodaty, H. (2018). Nexus between elder abuse, suicide, and assisted dying: the importance of relational autonomy and undue influence [online]. Macquarie Law Journal, 18, 79–92.

[ref33] White, N. , Kupeli, N. , Vickerstaff, V. and Stone, P. (2017). How accurate is the ‘Surprise Question’ at identifying patients at the end of life? A systematic review and meta-analysis. BMC Medicine, 15, 139. doi: 10.1186/s12916-017-0907-4 28764757 PMC5540432

[ref34] Williamson, V. , Murphy, D. and Greenberg, N. (2020). COVID-19 and experiences of moral injury in front-line key workers. Occupational Medicine (Lond), doi: 10.1093/occmed/kqaa052 PMC7184422 32239155

[ref35] World Health Organization (2020). Infection Prevention and Control Guidance for Long-Term Care Facilities in the Context of COVID-19: Interim Guidance 21 March 2020. Geneva, Switzerland: WHO Available at: https://apps.who.int/iris/bitstream/handle/10665/331508/WHO-2019-nCoV-IPC_long_term_care-2020.1-eng.pdf; accessed 10 April 2020.

[ref36] Zhou, F. et al. (2020). Clinical course and risk factors for mortality of adult inpatients with COVID-19 in Wuhan, China: a retrospective cohort study. Lancet, 395, 1054–1062. doi: 10.1016/S0140-6736(20)30566-3 32171076 PMC7270627

